# Modeling of Mesoscale Variability in Biofilm Shear Behavior

**DOI:** 10.1371/journal.pone.0165593

**Published:** 2016-11-02

**Authors:** Pallab Barai, Aloke Kumar, Partha P. Mukherjee

**Affiliations:** 1 Department of Mechanical Engineering, Texas A&M University, College Station, Texas, United States of America; 2 Department of Mechanical Engineering, University of Alberta, Edmonton, Alberta, Canada; University of Notre Dame, UNITED STATES

## Abstract

Formation of bacterial colonies as biofilm on the surface/interface of various objects has the potential to impact not only human health and disease but also energy and environmental considerations. Biofilms can be regarded as soft materials, and comprehension of their shear response to external forces is a key element to the fundamental understanding. A mesoscale model has been presented in this article based on digitization of a biofilm microstructure. Its response under externally applied shear load is analyzed. Strain stiffening type behavior is readily observed under high strain loads due to the unfolding of chains within soft polymeric substrate. Sustained shear loading of the biofilm network results in strain localization along the diagonal direction. Rupture of the soft polymeric matrix can potentially reduce the intercellular interaction between the bacterial cells. Evolution of stiffness within the biofilm network under shear reveals two regimes: a) initial increase in stiffness due to strain stiffening of polymer matrix, and b) eventual reduction in stiffness because of tear in polymeric substrate.

## Introduction

Bacteria can exist either as planktonic solutions or as surface associated colonies known as biofilms [1, 2]. The latter is now recognized to be dominant mode of bacterial life. Biofilms generally include several differentiated populations of bacterial cells that are embedded in a matrix of self-produced extracellular polymeric substances (EPS). EPS acts as natural ‘glue’ providing mechanical integrity to the biofilm structure [3]. Due to this social form of growth biofilm bacteria offers resilience to external stresses and thus provides itself with an existential advantage over planktonic form of living [4–7]. Biofilms have been implicated for their role in recurrent infections and biofouling [8, 9]. However, they also play a crucial role in several environmental processes including chemical cycles and waste-water treatment [10, 11]. Our need to control biofilm growth, as well as possess the ability to remove undesirable biofilms, necessitates a proper understanding of the material characteristics of this biological soft matter [12].

Biofilms are now recognized to belong to the class of viscoelastic materials; this behavior dictates deformation behavior of biofilms under shear forces [13]. In practical situations such behavior leads to clogging of biomedical devices [14], porous media [15, 16] as well as controls our ability to remove undesirable biofilms [17, 18]. Experimental investigation of the viscoelastic properties of biofilms have been performed using techniques such as capillary flow cells, rotating disk rheometry, holographic microrheology, microbead force spectroscopy and also by using deformable microfluidic devices[4, 5, 7, 19]. This has allowed researchers to quantify the elastic shear modulus as well as the relaxation time of the viscoelastic material. Interestingly, there exists a wide variability in experimental results even when performed for the same bacterial strain[5, 20, 21]. To elucidate these discrepancies, computational models have been developed to estimate the elastic modulus[22] and approximately understand the detachment of biofilms from its substrate[23, 24]. However, experiments have revealed interesting phenomena such as shear strain stiffening, which are nonlinear elastic response, are poorly understood even today in the context of biofilms[5, 6]. The adhesion strength of biofilms have also been observed to change both spatially and temporally[25]. Experimental evidence aside, a full understanding of biofilm properties remains elusive [6]. Heterogeneity and localization of cells and variability in EPS composition make rheological modeling of biofilms challenging[26–28]. Moreover, EPS contains large biological macromolecules whose unfolding behavior can introduce complex stress-strain relationships[29–32]. Recent modeling efforts have used fluid mechanics, network theory or finite element modeling to capture biofilm mechanical behavior[32–37]. It is imperative that open-ended questions such as those mentioned above be answered to move towards our goal of understanding biofilm deformation.

In the present article, a digital biofilm model (DBM) has been introduced that is developed using discrete lattice based methodologies. The lattice spring model has been used extensively in the domain of biomaterials and materials science to analyze the behavior of various complicated biomaterials and elastic brittle solids, respectively[38, 39]. In the present DBM, the entire biofilm is assumed to be a two phase media, which consists of rigid bacteria and soft deformable extracellular polymeric substrate (EPS). This model is developed by digitizing optical images of biofilm cross-sections and mapping a lattice spring network on top of it. Springs that lie inside the bacteria, display significantly large elastic modulus, whereas, the spring elements within the EPS matrix are more flexible in nature. It is assumed that, unfolding and subsequent stiffening of the polymeric chains (spring elements in this model) present within the EPS gives rise to the strain stiffening observed in biofilms[20, 29]. Further application of strain results in rupture of the polymer chain which gets manifested as fracture within the biofilm network[5, 30]. In this article, the authors attempt to capture the experimentally observed strain stiffening and subsequent rupture of the biofilm network under shear loading conditions[19]. To the best of our knowledge, this is the first work which attempts to model a biofilm using a lattice spring network and tries to explain the strain stiffening and subsequent softening of biofilm under externally applied load using the concept of EPS polymeric chain extension, stiffening and subsequent rupture. Along with capturing the strain-stiffening phenomena, this digital biofilm model is capable of analyzing the impact of different biofilm morphology, such as uniformly distributed or centrally clustered bacteria. Different bacterial cell morphology can also be studied using the present technique.

## Results

**Fig 1:**
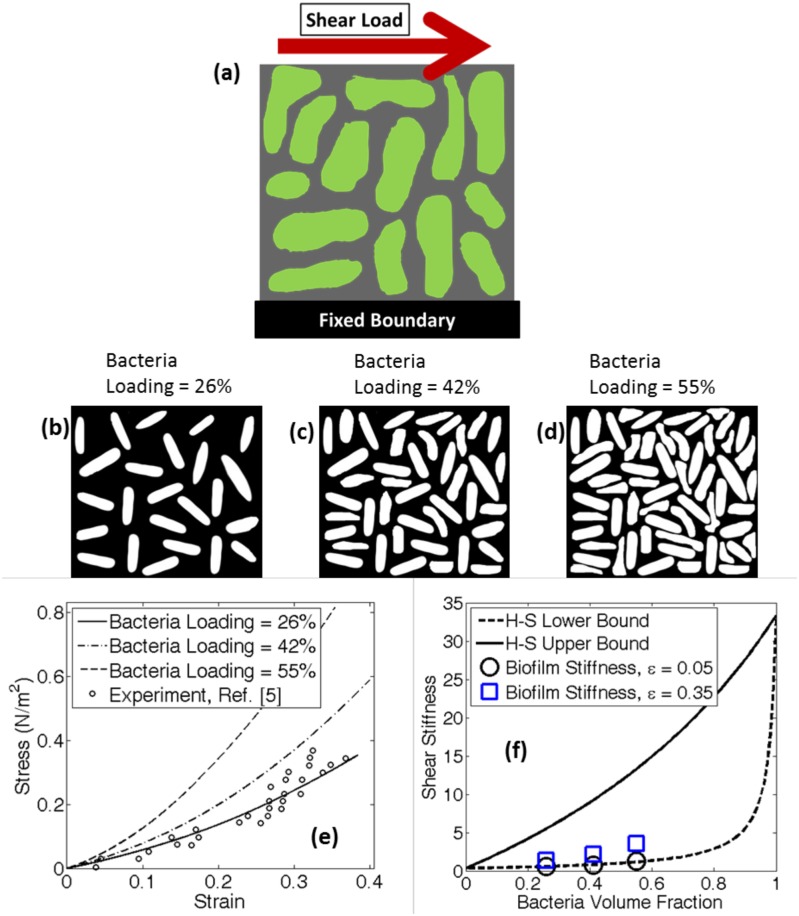
This figure shows different amount of bacteria loading and the corresponding stress—strain response. (a) A representative biofilm microstructure subjected to shear load. (b) Bacteria loading of 26% (white portion signifies bacteria and black signifies the EPS matrix). (c) Bacteria loading of 42%. (d) Bacteria loading of 55%. (e) Shear stress—strain response of the biofilm for the three types of bacteria loading scenario. Since the bacteria are considered as rigid objects, larger volume fraction of bacteria leads to higher stiffness for the biofilm. Stiffness for each lattice spring element in the EPS matrix has been set in such a way that the biofilm with 26% bacteria loading follow the experimental shear stress—strain data obtained from Stoodley et al (2002). (f) Based on the stiffness of the EPS matrix and the bacteria, Hashin—Shtrikman (H-S) lower and upper bounds of shear stiffness for the biofilm has been evaluated. Shear stiffness of the biofilm at 5% and 35% shear strain falls within the Hashin—Shtrikman bounds. The parameters used in these simulations are given in Table 1.

Using a unique lattice spring based digital biofilm model (DBM), strain-stiffening behavior observed in biofilm networks have been captured. Unfolding and subsequent stiffening of the protein chains located inside EPS resulted in increment of stiffness within the biofilm network. Even though there exist other polymeric materials within the EPS matrix that shows shear-thinning behavior for small time scales, only the shear-stiffening protein chains have been considered in the present study as a first approximation[40]. Since the bacteria located within the biofilm network acts as a rigid body, with increasing bacteria loading, effective stiffness of the biofilm network increases. The assumption of considering bacteria as a rigid object is based on prior experimental works by other researchers[41, 42]. In the present context, “bacteria loading” indicates the volume fraction of the biofilm domain occupied by living bacteria. In fact in the context of soft matter physics, rigid bacteria are often considered analogous to non-deformable colloidal particles[6]. While this assumption might not apply to all possible scenarios, we consider the domain space where this assumption is valid. Different portions of the same biofilm can have various bacteria loading. Fig 1(b), 1(c) and 1(d) shows three different cases of 26%, 42% and 55% bacteria loading, respectively. These experimentally observed images of biofilm belong to *Escherichia Coli 042* bacteria grown in the presence of an electric field, in a miniature culture chamber[43].

Under shear deformation, the stress-strain curve obtained for different bacterial loading has been shown in Fig 1(e). With increasing proportion of rigid bacteria, stiffness of the system increases. Strain stiffening is observed under all the three bacterial loading conditions. The spring stiffness parameter has been assigned in such a way that the stress-strain curve for the 26% bacteria loading corresponds to the experimental result reported by Stoodley et al. (2002) [5]. The exact parameters used for this comparison are reported in Table 1. Under externally applied shear load, the biofilms behave as elastic solid for a short period of time, and shows viscoelastic response at longer time intervals[5]. In the present context, only elastic response of the biofilm is being simulated. The stress-strain data-points adopted from Stoodley et al. were obtained by changing the externally applied load at a time interval of 5 seconds. The mechanical response at such short time intervals (with respect to the viscoelastic relaxation time scale of a biofilm) can be assumed to be elastic[13]. These biofilms were generated from *Pseudomonas aeruginosa* strains and grown under laminar flow conditions for 14 days with sufficient nutrient feed. No specific information was provided whether the biofilm being tested were at the beginning or end of life. According to the analysis reported in Schaudinn et al.[44], biofilms containing *Staphylococcus Epidermis* cultivated in static liquid cultures, that is 14 days old should be considered to have reached its end of life. The EPS matrix usually consists of polysaccharides, proteins, nucleic acids, phospholipids and other humic substances[45]. In the present context, it is assumed that the EPS matrix consists of polymeric protein chains that can uncoil under externally applied load.

**Table 1:** List of parameters used for the comparison with experimental results reported in Stoodley et al.[5] are provided below. The figure showing the strain-hardening behavior and comparison with experiments are provided in Fig 1(e).

Name of parameter	Unit	Value in EPS matrix	Value in rigid bacteria	Ref.
Young’s modulus	N/m^2^	1.0	100.0	[5]
Poisson’s ratio	—	0.33	0.33	—
Shear modulus	N/m^2^	0.33	33.33	[5]
Average energy threshold for strain-hardening	J/m^2^	10.0	—	[57, 58]
Energy threshold for sub-spring uncoiling (applicable to each spring)	J	1.25e-5±0.25e-5	—	—

It can be observed from Fig 1(e) that for higher bacteria loading, the strain stiffening starts earlier. An important point to note is that in our model, bacteria have been approximated as rigid particles [6]. Hence, under the application of an external force, majority of the deformation occurs within the EPS matrix because of its smaller stiffness. For the sections of biofilm with high bacteria loading also corresponds to low EPS content. All the strain stiffening comes only from the EPS matrix. To maintain equilibrium, all the portions of the biofilm should experience equal force. Due to the rigidity of bacteria, small deformation leads to large force. To produce similar amount of force in the EPS matrix, significant deformation has to occur. Hence, the springs adjacent to the bacteria enters the strain stiffening regime early enough. For biofilms containing large amount of EPS matrix, the strain gets equally distributed among all the protein chains, and strain stiffening initiates late in each of them. Hence, the biofilm microstructure that contains smaller proportion of EPS, experiences more strain, and the stiffening initiates earlier. On the other hand, polymeric substrates in biofilm microstructures containing a higher proportion of EPS are subjected to less strain, leading to delayed initiation of strain stiffening phenomena.

To check whether the stiffness values estimated using DBM is correct or not, a bound based analysis has been conducted in Fig 1(f). The Hashin-Shtrikman (H-S) upper and lower bounds for shear modulus were plotted along with stiffness of the biofilm for all the three different bacteria loading at 5% and 35% shear strain [46]. The exact expressions of Hashin-Shtrikman bounds have been estimated by using a variational approach and applicable to any random geometry of the bacteria inclusions. The Hashin-Shtrikman bound has been used to verify the biofilm elastic modulus predicted by DBM. The lower and upper bounds of the shear modulus are denoted by *G*_*l*_ and *G*_*u*_, respectively. Their expressions are given as follows:

$$G_{l}=G_{1}+c_{2}\cdot {\left[\frac{1}{G_{2}-G_{1}}+\frac{6\left(K_{1}+2G_{1}\right)\cdot c_{1}}{5G_{1}\cdot \left(3K_{1}+4G_{1}\right)}\right]}^{-1}$$ (1)

and

$$G_{u}=G_{2}+c_{1}\cdot {\left[\frac{1}{G_{1}-G_{2}}+\frac{6\left(K_{2}+2G_{2}\right)\cdot c_{2}}{5G_{2}\cdot \left(3K_{2}+4G_{2}\right)}\right]}^{-1}$$ (2)

Here, *G* represents shear modulus, *K* represents bulk modulus and *c* indicates the volume fraction. The subscript “1” and “2” signifies properties of the EPS matrix and the bacteria, respectively. The stiffness at 5% strain corresponds to the lower bound, whereas; at 35% strain the shear stiffness lie perfectly inside the H-S bounds. This implies that the H-S bounds for shear stiffness are applicable in biofilm networks as well for the purpose of checking the validity of experimental results. These H-S bounds are applicable to both 2D and 3D two-phase media. Since the 2D biofilm geometries simulated here are plane strain approximations of a 3D geometry with zero stress along the out-of-plane direction, the H-S bounds are applicable. In the present 2D analysis, it is assumed that similar volume fraction and morphology of bacteria is observed along the out-of-plane direction of the biofilm. Under these conditions, the 2D analysis reported here is capable of capturing the 3D response of the realistic biofilms. However, if the volume fraction or morphology of bacteria changes along the out-of-plane direction, approximation of the entire 3D biofilm by a 2D representation is not possible.

**Fig 2:**
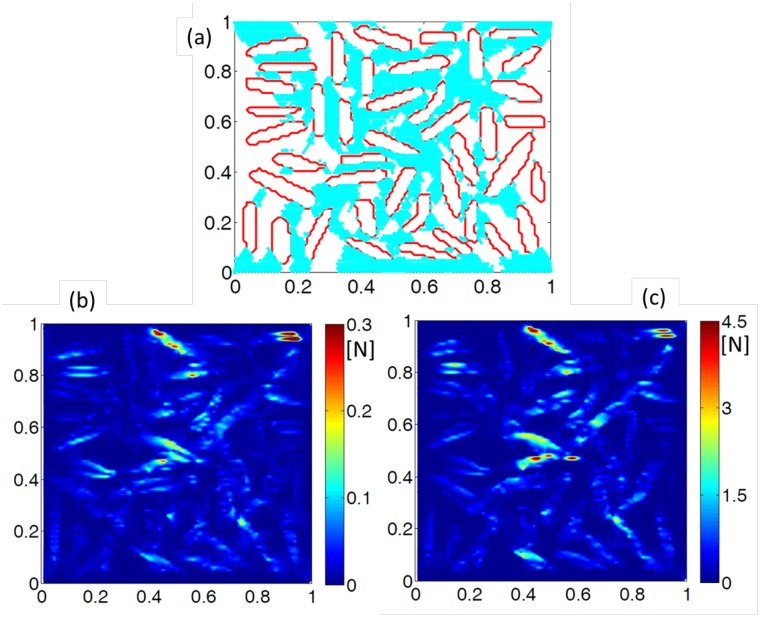
Force contours depict the corresponding regions which experience strain hardening. No rupture has been considered in this simulation. (a) The cyan region signifies the portion which has undergone strain hardening behavior. Under shear loading, maximum shear strain has been observed along the diagonal directions. This is equivalent to the formation of shear bands observed in conventional solid materials. (b) Force contour observed at 5% shear strain. The magnitude of force is in the order of 0.3N. (c) Force contour observed at 35% shear strain. Magnitude of force is around 4.5N, which is an order of magnitude greater than the forces observed under 5% strain. Larger amount of forces accumulate along diagonal directions, which eventually leads to enhanced strain hardening along the diagonal direction. Similar behavior has been reported in part (b) of this image. The parameters used in these simulations are given in Table 1.

It is conjectured that strain stiffening occurs within biofilm networks because of the unfolding of protein chains. The question is whether the entire EPS matrix experiences uniformly distributed shear strain, or localization of shear strain occurs within the DBM network. The biofilm morphology taken into consideration consists of 42% bacterial loading with uniform distribution (also described in the methodology section). Fig 2(a) shows (in cyan) the location of those springs that has experienced unfolding. It has been observed that very small amount of unfolding occurs on the left and right side of the biofilm network. Majority of the strain got localized along the diagonal direction, which led to unfolding of springs along the diagonal. This type of shear strain localization along the diagonal has also been observed in metals [47]. From this analysis, it is evident that unfolding of the protein chains is localized along the diagonal of the EPS matrix. Rupture behavior has not been simulated in this case. The parameters adopted to run the simulations provided in Fig 2(a), 2(b) and 2(c) are provided in Table 1.

Force contours observed inside the biofilm network have been plotted in Fig 2(b) and 2(c) under externally applied strains of 5% and 35%, respectively. For 5% strain, small amount of force has been observed within the network, which has a maximum value of 0.3N. As the strain is increased to 35%, large amount of force is generated, which shows a maximum value of 4.5N. It is clear that seven fold increment in strain (5% to 35%) results in fifteen-fold increase in the magnitude of force (0.3N to 4.5N). This nonlinearity is attributed to the strain stiffening behavior observed due to the unfolding of EPS protein chains. Since unfolding of sub-springs lead to stiffening of the springs, an unfolded spring produces larger force. Thus, the unfolded springs along the diagonal direction observed in Fig 2(a) naturally leads to localization of force along the diagonal (Fig 2(b) and 2(c)).

**Fig 3:**
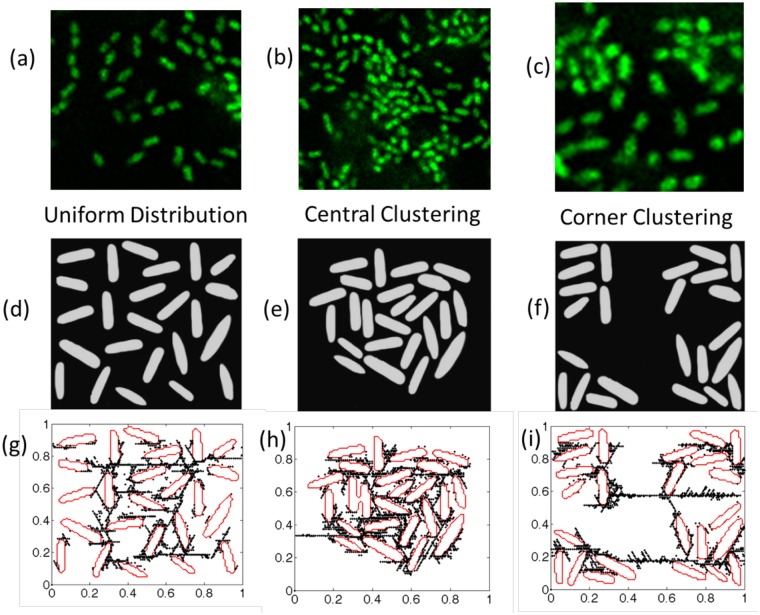
Different clustering of bacteria inside the biofilm along with the rupture patterns are displayed here. (a) Uniform distribution of bacteria within a biofilm. (b) Clustering of bacteria in the central portion of the domain. (c) Clustering of bacteria is observed around the corner of the domains. (d) Schematic representation of uniformly distributed bacteria inside biofilms. (e) Schematic representation of central clustering observed in biofilms. (f) Schematic representation of corner clustering within biofilms. (g) Distribution of rupture within the uniformly distributed bacteria. When all the bacteria are well dispersed, damage in the biofilm is not localized enough for an entire bacterium to be completely detached from the EPS matrix. (h) Rupture profile as observed for centrally clustered bacteria. When the bacteria are clustered within the biofilm, force concentration is very high around the bacterium, which leads to mechanical detachment of the EPS matrix from certain bacteria. (i) Evolution of damage for corner clustering scenario. Purpose of the corner clustering is to investigate how the tear would propagate through the EPS between two clusters of bacteria. It can be concluded that cracks through EPS matrix propagates to the nearest cluster. There is very little possibility for a tear through EPS to span between two clusters which are located far away from each other. The parameters are given in Table 2.

None of the simulations discussed till now had rupture of the springs incorporated within the DBM. The biofilm microorganisms (such as, bacteria) are embedded within the EPS matrix which is composed mostly of polysaccharides, proteins and lipids[48]. As rupture of springs is added into the simulations, the EPS matrix may experience detachment from the bacteria. The EPS matrix interacts with the environment by attaching biofilms to a surface. Sorption and transport of dissolved and particulate substances from the environment provides nutrients for biofilm organisms[44, 45, 48]. Rupture of the EPS matrix can affect the chemical communication within the bacteria, which has the potential to impact the lifespan of a biofilm. In a nutrient poor condition, the bacteria may escape to the planktonic phase. By incorporating rupture of the protein chains, estimation will be made about the conditions when tearing can appear inside the EPS matrix. To simulate various bacterial morphologies, we generated various bacterial flocculation based on experimental images[43], which were later digitized. We considered various scenarios, such as, bacteria can be uniformly distributed (as shown in Fig 3(a) and 3(d)) or extremely clustered at the center (see Fig 3(b) and 3(e)). A third case of corner clustering has also been considered here to analyze the propagation of tear within the EPS matrix from one cluster to the other (see Fig 3(c) and 3(f)). Generation and nucleation of microcracks within uniformly distributed, centrally clustered and corner clustered bacteria are shown in Fig 3(g), 3(h) and 3(i), respectively. For all the simulations involving rupture of biofilms, the elastic and strength parameters reported in Table 2 have been adopted.

**Table 2:** List of parameters used for comparison with rupture experiments reported in Korstgens et al.[19] are provided below. Figure showing the rupture behavior and comparison with experimental results are provided Fig 4(a).

Name of parameter	Unit	Value in EPS matrix	Value in rigid bacteria	Ref.
Young’s modulus	N/m^2^	8000.0	800000.0	[19]
Poisson’s ratio	—	0.33	0.33	—
Shear modulus	N/m^2^	3000.0	300000.0	[19]
Average threshold for strain hardening	J/m^2^	2.0	—	[57, 58]
Average threshold for EPS rupture	J/m^2^	7.0	—	[57, 58]
Energy threshold for sub-spring uncoiling (applied to each spring)	J	1.05e-3±0.05e-3	—	—
Energy threshold for spring rupture (applied to each spring)	J	4.0e-3	—	—

Density of ruptured protein chains within the EPS matrix is relatively less for the uniform distribution (see Fig 3(g)). As a result some portion of every bacteria are connected with the EPS matrix. On the other hand, the biofilm morphology with centrally clustered bacteria experiences larger amounts of rupture inside the EPS matrix adjacent to the bacteria (see Fig 3(h)). Extreme amount of tear within EPS has the potential to completely detach some of the bacteria from the biofilm network, which can impact their chemical communication. The amount of rupture and tear in the EPS matrix within uniformly distributed and clustered biofilm seems minimal. However a thorough analysis clearly indicates that several bacteria within the clustered sample (shown in Fig 3(h)) is completely detached from the EPS matrix and suffers due to lack of nutrients. All the bacteria are connected to the EPS matrix for the uniform distribution (shown in Fig 3(g)). Thus, high clustering of bacteria can be beneficial for removal of biofilm through the application of mechanical force. The physical reason behind observation of enhanced rupture in high clustering, stems from the fact that regions with high bacterial loading also experience lower concentration of EPS. Majority of the externally applied strain is accommodated by the soft EPS matrix. Strain stiffening is observed earlier in regions with less amount of polymeric substrate, which subsequently leads to rupture. This phenomenon is similar to that observed in Fig 1(e).

Due to the presence of larger volume fraction of EPS matrix adjacent to each bacterium, less strain stiffening and rupture occurs under uniform distribution. The corner clustering case has been investigated to understand the propagation of tear through the EPS matrix in between two clusters. It is evident from Fig 3(i) that the cracks through the EPS matrix propagates from one cluster to the nearest neighboring cluster. Probability of propagation of tear along the diagonal direction is relatively less. Rupture propagation is a mechanism to release strain energy. Connecting the ruptures in the nearest neighboring clusters provides a shorter pathway to release strain energy quickly. That is why no rupture patterns have been observed which connects diagonally opposite clusters.

**Fig 4:**
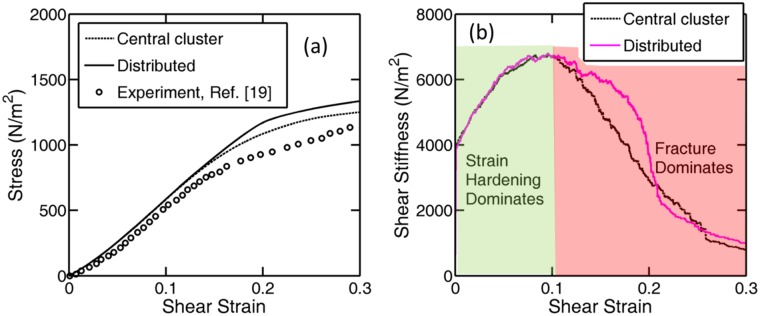
Evolution of stress and stiffness for a biofilm under shear loading. (a) Shear stress is plotted against shear strain. For biofilms with bacterial clustering, rupture initiates at a stress level of approximately 1100N/m^2^. Whereas, for biofilms with uniformly dispersed bacteria, rupture initiates at a higher stress magnitude, around 1300 N/m^2^. (b) Evolution of shear stiffness of the biofilm under increasing shear strain. Initially, because of strain hardening, stiffness of the biofilm increases. But after sometime, rupture starts to dominate and the overall stiffness of the biofilm drops. The two regions are clearly demonstrated in the figure, where, strain hardening dominates initially and rupture takes over towards the end. Reduction in stiffness starts earlier for biofilms with central clustering. This means, it is easier to tear the biofilms with clustering as compared to biofilms with uniformly distributed bacteria. The parameters used in these simulations are given in Table 2.

Stress vs. strain response of the biofilm network under shear strain induced loading has been shown in Fig 4(a) using the parameters reported in Table 2. Mechanical rupture has also been incorporated within the simulation. Due to unfolding of springs shear stiffness increases initially. Once the rupture of EPS matrix is initiated, stiffness of the network decreases significantly. Stress-strain curve for both the central clustering and uniformly distributed bacteria loading has been shown. The result obtained from computational analysis corresponds very closely with that observed in experiments conducted by Korstgens *et al*. (see Fig 4(a))[19]. Because of localized strain stiffening, early initiation of rupture has been observed in the central cluster. For this particular case, cracks initiate at a stress level of 1100N/m^2^. For uniformly distributed bacteria loading, rupture initiation starts at a stress level of 1300N/m^2^. Delay in microcrack nucleation happens because of the fact that uniformly distributed bacteria contains large amount of EPS matrix adjacent to it, which acts as a buffer against rupture initiation.

Evolution of shear stiffness has been plotted in Fig 4(b) with respect to increasing shear strain. It has been observed that strain stiffening dominates till a particular strain. Because of the presence of tear in the protein chains of EPS matrix, rupture behavior starts to dominate after that critical strain. Two regions where strain stiffening dominates, and where rupture dominates have been clearly marked in Fig 4(b). For the case of central clustering, domination of rupture over strain stiffening starts earlier than the case of uniformly distributed bacteria. This early initiation of fracture in the EPS matrix is attributed to the fact that in centrally clustered biofilm, very little amount of EPS matrix exists adjacent to the bacteria. Under shear-induced deformation, the EPS matrix carries majority of the strain and experiences early initiation of strain stiffening and subsequent rupture in the regions with less amount of the polymeric substrate. As a result, the uniformly distributed bacteria experiences delayed initiation of strain stiffening and rupture due to the presence of excessive amount of EPS matrix around each bacterium.

## Discussion

In our effort to create the DBM framework, we have made certain simplifying assumptions. For example, the biofilm has been assumed to be a two phase system, soft EPS matrix and rigid bacteria. However, in a realistic situation EPS matrix can contain inert biomass, water and/or air filled voids[5, 19, 49–51]. As a result, practical situations are quite complex and a full model has to incorporate these different aspects. Void spaces filled with air would be of paramount interest in biofilms that are partially exposed to air (such as those grown on Agar plates). In the present context, images that motivated the DBM model were taken from biofilms that are totally immersed in a liquid environment and hence air-like voids have not been considered[43]. It is assumed that the biofilm had sufficient time to equilibrate with its aqueous environment and hence the EPS/water network structure could be considered to be a homogenous system. Extending the current computational methodology to such complex situations is indeed possible, but would require extensive validation and is left as an exercise for a future work.

Morphology of the bacteria cells significantly impact the overall viscoelastic and dynamic behavior of biofilms[37]. Another simplifying assumption worth mentioning is the fact that effect of bacterial cell morphology has been neglected in the present study. However, the shape of the cells adopted here has been inspired by experimentally observed images of biofilms[43]. Since the protein chains within the EPS matrix mostly govern the strain-stiffening behavior, bacterial morphology has minimum impact there. Hence the effect of cell morphology can be neglected as a first approximation. Detailed analysis of the shape of the cells will be conducted in future.

While it is known that biofilms can exhibit complex viscoelastic response to external forces, only elastic response has been modeled here. This is equivalent to considering a situation where the response time (*t*) is much smaller than the viscoelastic relaxation time-scale (*λ*). Some studies indicate that biofilms can be characterized by a dominant viscoelastic time scale of 20 minutes[52] and hence considering a scenario where *t* << *λ* as elastic, is quite realistic. Moreover, viscoelastic responses are often linked to very large deformations[53], which is outside of the scope of the current investigation.

## Conclusion

In this article, a novel digital biofilm model has been developed, which has the potential to take into consideration the effect of any bacteria–EPS microstructure observed in real biofilms. Different volume fractions of bacterial cells show that for higher bacteria loading, stiffer stress-strain response is obtained. Also, under high bacteria loading enhanced strain hardening occurs within EPS. It leads to earlier initiation of tears inside the protein chains. Similar to strain localization observed in metals, localized strain stiffening along the diagonal direction has been observed in biofilm networks under shear deformation. Enhanced rupture has been observed in EPS matrix that is adjacent to the bacteria in a closely packed cluster. As a result, detachment of the bacterial cells from the EPS matrix is possible in a highly clustered domain under mechanically induced shear type load. By plotting the evolution of stiffness in a biofilm network, two different regions have been recognized. In the first domain strain stiffening dominates and the effective stiffness of the biofilm network increases. In the second region, the effect of rupture of the polymer matrix plays a major role and eventually results in reduction of the effective stiffness.

As a living system biofilm adapts with time and its biological activity is intricately tied to its mechanical and transport behavior. Most of these are currently neglected as we have considered only the short-time scale elastic strain-hardening and rupture response of biofilms to external shear. This implies that the time-scale of shear (*τ*) is much smaller than the relaxation time scale of the biofilm (*λ*), i.e. *τ* << *λ*. It has been reported that *λ* ~ 20 minutes for many different biofilm species[52]. Hence for shear forces lasting a short time, it is an appropriate first approximation to neglect growth and other biological activities[13]. Elastic rupture of the EPS matrix in the shorter time scale allows for the viscoelastic movement of biofilms in the longer time range. Naturally, for simulating much longer time scales the assumption of linear elastic behavior would not remain valid.

## Methodology

**Fig 5:**
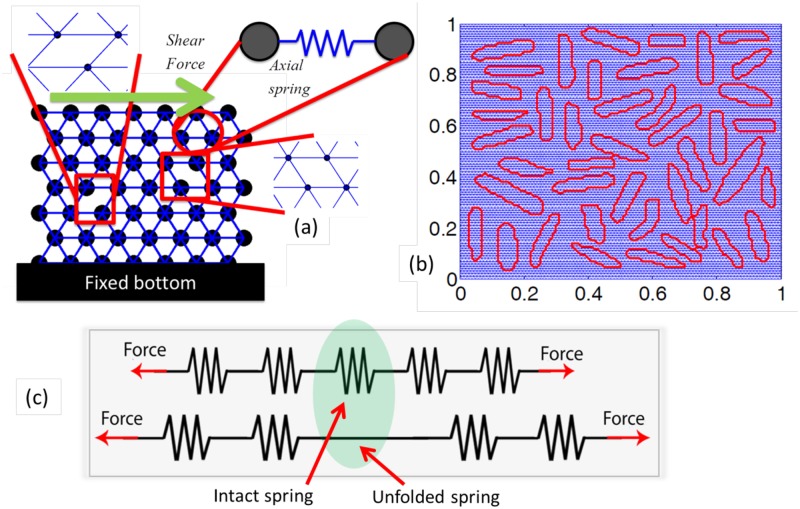
These figures demonstrate a schematic diagram of the lattice spring network, digitization of the biofilm domain and a schematic representation of the unfolding of springs. (a) The lattice spring network consists of a triangular mesh of spring elements. Each spring shows only axial stiffness. The mass remains concentrated at each node. The bottom of the network is fixed and shear force is applied at the top. (b) The digitized biofilm domain is displayed here. The red boundaries signify the boundaries for each of the bacteria. Blue portion is the mesh used to discretize the biofilm domain. (c) A schematic representation of the unfolding of springs. Under externally applied load, as the energy in a spring exceeds its unfolding threshold (the spring in the middle), it becomes straight and displays an infinite stiffness.

In this article, morphology aware computational model has been developed that can elucidate the shear behavior of a biofilm. Here the word “morphology aware” indicates the fact that the developed model can simulate any morphology of the bacteria cells as well as different clustering of bacteria within the biofilm (such as, uniformly distributed or clustered presence of bacteria cells). In order to investigate the effect of shear force on a biofilm (Fig 1(a)), we begin by using experimental visualization of the biofilm structure to create a digital representation of the biofilm (Fig 1(b)–1(d)). Once the digital representation of the biofilm is complete, then a single domain modeling approach is applied by superimposing the digitized biofilm microstructure on top of a network of lattice springs (see Fig 5(b)). Here the word “single modeling approach” indicates that only a single mesh has been generated. Different regions of the mesh has been assigned different properties based on the whether it belongs to a “bacteria” or “EPS matrix”. This computational characterization of a realistic biofilm microstructure has been named as Digital Biofilm Model (DBM).

As shown in Fig 5(a), in the digital biofilm model a triangular network of lattice springs has been considered to analyze the mechanical deformation of the biofilm. In the quasistatic computational scheme adopted in the current research, entire mass of the system is assumed to be concentrated at uniformly distributed nodes [39]. Any two adjacent nodes are connected by a spring, which shows only axial resistance. The triangular spring network leads to a coordination number of six. Interaction with only the nearest neighbors has been taken into consideration. Because of the triangular configuration, Poisson’s ratio of 1/3 has been observed for this network.

Superimposing the digitized biofilm on top of the triangular network, springs that lie within the bacteria and springs that reside inside the EPS matrix has been separated (see Fig 5(b)). In order to estimate stiffness of the biofilm network, the bacteria were assumed to be rigid with respect to the extracellular polymeric substrate (EPS) [6]. Furthermore, we restrict ourselves to studying biofilm response to shear forces for time scales much smaller than the viscoelastic relaxation time scales (i.e. *t* << *λ*) [5]. The elastic behavior dominates biofilm response in such small time scale. The EPS matrix consists of protein chains, which show strain-stiffening behavior due to unfolding of the polymer-chains[30]. Since the biofilm consists of soft polymeric gels, due to external effects the film can go through large deformations. Sometime shear strains as large as 50% can be observed because of flow induced force [7].

The rules applied to the spring are: (a) springs which are representative of bacteria have a very high spring constant [6], (b) springs which are in the EPS have a low spring constant and an energy prescribed limit is imposed. When the threshold strain energy is exceeded the springs become ‘uncoiled’ and are represented by a higher magnitude of spring constant. This latter rule is inspired by findings of Buehler [29, 30]. Using molecular dynamic simulations, unfolding of the helical patterns were captured which leads to stiffening of the entire polymer chain. The untangling mechanism was initiated by the stutter defect present within a protein chain. Once all the protein chains go through the unfolding mechanism, the extended fiber show relatively high stiffness, but cannot sustain a significant rise in externally applied strain. (c) Once the strain energy stored in a spring exceeds its rupture threshold, the spring breaks. It is irreversibly removed from the network of lattice springs and the force carried by this spring gets distributed among its neighbors [54].

To capture the unfolding of springs, it is assumed that each spring in the network consists of eight folded sub-springs [32, 55, 56]. Due to application of shear strain, as each of the sub-springs unfold stiffness of the full spring increases (see Fig 5(c)). After the eighth sub-spring has unfolded, application of more external load leads to rupture of the entire spring [38]. The remaining question is how to determine when the unfolding of sub-springs will occur. An energy prescribed criteria has been adopted in this study. Under externally applied loads, the local force (***f***) and displacement (***u***) has been calculated which equilibrate the spring network. Strain energy in each spring is calculated as, *ψ* = (1/2)***f***·***u***. With increasing amount of externally applied load, the total strain energy stored in each spring increases. Eight uniformly distributed random energy thresholds have been generated for each spring (*ψ*_*t*,*i*_;*I* = 1,2,3,…,8) in such a way that *ψ*_1_ < *ψ*_2_ < *ψ*_3_ <…< *ψ*_8_. As the strain energy in the full spring exceeds an unfolding threshold (*ψ* > *ψ*_*t*,*i*_), one sub-spring unfolds and the stiffness of the full spring is increased (shown in Fig 5(c)). This untangling mechanism continues until all the eight sub-springs have unfolded. Stiffness of the spring reaches its maximum value at that point [30].

Another energy threshold has been assigned to all the springs that correspond to its ultimate strength (*ψ*_*t*,*u*_) and it follows the constraint *ψ*_*t*,*u*_ > *ψ*_8_. Once the strain energy in the completely unfolded spring exceeds its ultimate rupture threshold (*ψ > ψ*_*t*,*u*_), the spring is assumed to be broken. Hence, it is irreversibly removed from the lattice network and the contribution of the spring is completely eliminated from the stiffness matrix. Rupture in the lattice spring network results in reduction in stiffness of the entire biofilm. Since the spring constant of the full spring increases with each unfolding of the sub-springs, the stiffness of the lattice network also changes. In order to correctly capture the increase in stiffness and model the spring deformation accordingly, an iterative technique has been adopted. The externally applied load is increased in increments of very small magnitude. All the sub-springs that unfold or full springs that break within a loading interval are modified accordingly. The energy in each springs are also estimated in increments, *ψ*^*n+*1^ = *ψ*^*n*^ + (1/2)Δ***f***·Δ***u***. Here, *ψ*^*n+*1^ and *ψ*^*n*^ signifies the strain energy at the current and the previous steps, respectively. Δ***f*** and Δ***u*** corresponds to the incremental force and displacement, respectively, observed in the current loading interval.

Estimation of the correct parameters of the EPS matrix and the rigid bacteria that can successfully predict the strain hardening and rupture behavior of the biofilms is a challenging task. However, the elastic behavior, in terms of stiffness, strain hardening and rupture, of an entire biofilm has been reported in some of the recently published articles[5, 6, 19]. For the simulations reported in this article, the elastic properties of the EPS matrix and rigid bacteria have been estimated such that the overall biofilm response closely follows the experimentally observed pattern. Exact values of the parameters adopted for the comparison with strain hardening and rupture behavior of biofilms are provided in Tables 1 and 2, respectively. The main purpose of this article is to demonstrate the basic physics behind the strain hardening and subsequent softening observed in biofilms. The range of parameters adopted to study these effects does not alter the physical explanation. Hence, usage of highly varying magnitude of parameters is well justified. Detailed discussion regarding the mathematics behind the computational methodology and a study of mesh size dependence has been incorporated within the Supplementary section (see S1 File).

## Supporting Information

S1 File Modeling of Mesoscale Variability in Biofilm Shear Behavior (Supplementary information). This file contains detailed derivation of the digital biofilm model (DBM) and its grid independence study.
(DOCX)

## References

[1] Renner LD, Weibel DB. Physicochemical regulation of biofilm formation. Mrs Bull. 2011;36(5):347–55. 10.1557/Mrs.2011.65. WOS:000293237700012. 22125358 PMC3224470

[2] Wong GCL, O'Toole GA. All together now: Integrating biofilm research across disciplines. Mrs Bull. 2011;36(5):339–45. 10.1557/Mrs.2011.64. WOS:000293237700011. 24465088 PMC3899391

[3] Flemming HC, Wingender J. The biofilm matrix. Nat Rev Microbiol. 2010;8(9):623–33. 10.1038/Nrmicro2415. WOS:000280855500009. 20676145

[4] Klapper I, Rupp CJ, Cargo R, Purvedorj B, Stoodley P. Viscoelastic fluid description of bacterial biofilm material properties. Biotechnology and Bioengineering. 2002;80(3):289–96. 10.1002/bit.10376 12226861

[5] Stoodley P, Cargo R, Rupp CJ, Wilson S, Klapper I. Biofilm material properties as related to shear-induced deformation and detachment phenomena. J Ind Microbiol Biotech. 2002;29(6):361–7. 10.1038/sj.jim.7000282 12483479

[6] Wilking JN, Angelini TE, Seminara A, Brenner MP, Weitz DA. Biofilms as complex fluids. Mrs Bull. 2011;36(5):385–91. 10.1557/Mrs.2011.71. WOS:000293237700017.

[7] Aggarwal S, Hozalski RM. Effect of Strain Rate on the Mechanical Properties of Staphylococcus epidermidis Biofilms. Langmuir. 2012;28(5):2812–6. 10.1021/La204342q. WOS:000299864500061. 22217007

[8] Khoo X, Grinstaff MW. Novel infection-resistant surface coatings: A bioengineering approach. Mrs Bull. 2011;36(5):357–66. 10.1557/Mrs.2011.66. WOS:000293237700013.

[9] Shrout JD, Tolker-Nielsen T, Givskov M, Parsek MR. The contribution of cell-cell signaling and motility to bacterial biofilm formation. Mrs Bull. 2011;36(5):367–73. 10.1557/Mrs.2011.67. WOS:000293237700014. 22053126 PMC3204577

[10] Mohan SV, Rao NC, Sarma PN. Composite chemical wastewater treatment by biofilm configured periodic discontinuous batch process operated in anaerobic metabolic function. Enzyme Microb Tech. 2007;40(5):1398–406. 10.1016/j.enzmictec.2006.10.023. WOS:000245519000058.

[11] Nealson KH, Finkel SE. Electron flow and biofilms. Mrs Bull. 2011;36(5):380–4. 10.1557/Mrs.2011.69. WOS:000293237700016.

[12] Karimi A, Karig D, Kumar A, Ardekani AM. Interplay of physical mechanisms and biofilm processes: review of microfluidic methods. Lab Chip. 2015;15(1):23–42. 10.1039/C4lc01095g. WOS:000346478100003. 25385289 PMC4261921

[13] Das S, Kumar A. Formation and post-formation dynamics of bacterial biofilm streamers as highly viscous liquid jets. Sci Rep-Uk. 2014;4 Artn 7126 10.1038/Srep07126. WOS:000346177800006. 25410423 PMC4237988

[14] Drescher K, Shen Y, Bassler BL, Stone HA. Biofilm streamers cause catastrophic disruption of flow with consequences for environmental and medical systems. P Natl Acad Sci USA. 2013;110(11):4345–50. 10.1073/pnas.1300321110. WOS:000316238300044. 23401501 PMC3600445

[15] Kim JW, Choi H, Pachepsky YA. Biofilm morphology as related to the porous media clogging. Water Res. 2010;44(4):1193–201. 10.1016/j.watres.2009.05.049. WOS:000275551300017. 19604533

[16] Valiei A, Kumar A, Mukherjee PP, Liu Y, Thundat T. A web of streamers: biofilm formation in a porous microfluidic device. Lab Chip. 2012;12(24):5133–7. 10.1039/C2lc40815e. WOS:000311964700002. 23123600

[17] Simoes M, Simoes LC, Vieira MJ. A review of current and emergent biofilm control strategies. Lwt-Food Sci Technol. 2010;43(4):573–83. 10.1016/j.lwt.2009.12.008. WOS:000275013700001.

[18] Kumar A, Karig D, Acharya R, Neethirajan S, Mukherjee PP, Retterer S, et al Microscale confinement features can affect biofilm formation. Microfluid Nanofluid. 2013;14(5):895–902. 10.1007/s10404-012-1120-6. WOS:000318489500013.

[19] Korstgens V, Flemming HC, Wingender J, Borchard W. Uniaxial compression measurement device for investigation of the mechanical stability of biofilms. J Microbiol Meth. 2001;46(1):9–17. 10.1016/S0167-7012(01)00248-2. WOS:000169339400002. 11412909

[20] Hohne DN, Younger JG, Solomon MJ. Flexible Microfluidic Device for Mechanical Property Characterization of Soft Viscoelastic Solids Such as Bacterial Biofilms. Langmuir. 2009;25(13):7743–51. 10.1021/La803413x. WOS:000267533800082. 19219968 PMC2723186

[21] Storm C, Pastore JJ, MacKintosh FC, Lubensky TC, Janmey PA. Nonlinear elasticity in biological gels. Nature. 2005;435(7039):191–4. 10.1038/Nature03521. WOS:000229021100036. 15889088

[22] Aravas N, Laspidou CS. On the calculation of the elastic modulus of a biofilm streamer. Biotechnology and Bioengineering. 2008;101(1):196–200. 10.1002/Bit.21865. WOS:000258866800020. 18383138

[23] Picioreanu C, van Loosdrecht MCM, Heijnen JJ. Effect of diffusive and convective substrate transport on biofilm structure formation: A two-dimensional modeling study. Biotechnology and Bioengineering. 2000;69(5):504–15. 10.1002/1097-0290(20000905)69:5<504::Aid-Bit5>3.0.Co;2-S. WOS:000088838100005. 10898860

[24] van Loosdrecht MCM, Heijnen JJ, Eberl H, Kreft J, Picioreanu C. Mathematical modelling of biofilm structures. Anton Leeuw Int J G. 2002;81(1–4):245–56. 10.1023/A:1020527020464. WOS:000178390200024. 12448723

[25] Ohashi A, Harada H. Adhesion Strength of Biofilm Developed in an Attached-Growth Reactor. Water Sci Technol. 1994;29(10–11):281–8. WOS:A1994PU77600034.

[26] Bishop PL, Zhang TC, Fu YC. Effects of Biofilm Structure, Microbial Distributions and Mass-Transport on Biodegradation Processes. Water Sci Technol. 1995;31(1):143–52. 10.1016/0273-1223(95)00162-G. WOS:A1995RD27100014.

[27] Zhang TC, Bishop PL. Density, Porosity, and Pore Structure of Biofilms. Water Res. 1994;28(11):2267–77. 10.1016/0043-1354(94)90042-6. WOS:A1994PJ23800004.

[28] Zhang TC, Fu YC, Bishop PL. Competition in Biofilms. Water Sci Technol. 1994;29(10–11):263–70. WOS:A1994PU77600032.

[29] Buehler MJ. Hierarchical chemo-nanomechanics of proteins: Entropic elasticity, protein unfolding and molecular fracture. J Mech Mater Struct. 2007;2(6):1019–57. 10.2140/Jomms.2007.2.1019. WOS:000256406600004.

[30] Buehler MJ, Ackbarow T. Fracture mechanics of protein materials. Materials Today. 2007;10(9):46–58. 10.1016/S1369-7021(07)70208-0

[31] Ghosh R, Kumar A, Vaziri A. Type-IV Pilus Deformation Can Explain Retraction Behavior. Plos One. 2014;9(12). ARTN e114613 10.1371/journal.pone.0114613. WOS:000346375400040. 25502696 PMC4264779

[32] Ehret AE, Bol M. Modelling mechanical characteristics of microbial biofilms by network theory. J R Soc Interface. 2013;10(78). ARTN 20120676 10.1098/rsif.2012.0676. WOS:000311939400025. 23034354 PMC3565799

[33] Duddu R, Bordas S, Chopp D, Moran B. A combined extended finite element and level set method for biofilm growth. Int J Numer Meth Eng. 2008;74(5):848–70. 10.1002/nme.2200. WOS:000255730100007.

[34] Horn H, Lackner S. Modeling of Biofilm Systems: A Review. Adv Biochem Eng Biot. 2014;146:53–76. 10.1007/10_2014_275. WOS:000348637900003. 25163572

[35] Laspidou CS, Rittmann BE, Karamanos SA. Finite element modelling to expand the UMCCA model to describe biofilm mechanical behavior. Water Sci Technol. 2005;52(7):161–6. WOS:000233557400025.

[36] Rudge TJ, Steiner PJ, Phillips A, Haseloff J. Computational Modeling of Synthetic Microbial Biofilms. Acs Synth Biol. 2012;1(8):345–52. 10.1021/sb300031n. WOS:000307697900005. 23651288

[37] Storck T, Picioreanu C, Virdis B, Batstone DJ. Variable Cell Morphology Approach for Individual-Based Modeling of Microbial Communities. Biophys J. 2014;106(9):2037–48. 10.1016/j.bpj.2014.03.015. WOS:000335610100026. 24806936 PMC4017289

[38] Nukala PKVV, Simunovic S. A continuous damage random thresholds model for simulating the fracture behavior of nacre. Biomaterials. 2005;26(30):6087–98. 10.1016/j.biomaterials.2005.03.013. WOS:000230538700018. 15958244

[39] Zhao GF, Fang JN, Zhao J. A 3D distinct lattice spring model for elasticity and dynamic failure. Int J Numer Anal Met. 2011;35(8):859–85. 10.1002/Nag.930. WOS:000290443300001.

[40] Lembre P. Exopolysaccharides of the Biofilm Matrix: A Complex Biophysical World In: Karunaratne DN, editor. The Complex World of Polysaccharides: INTECH; 2012.

[41] Arnoldi M, Fritz M, Bauerlein E, Radmacher M, Sackmann E, Boulbitch A. Bacterial turgor pressure can be measured by atomic force microscopy. Phys Rev E. 2000;62(1):1034–44. 10.1103/PhysRevE.62.1034. WOS:000088274200034. 11088560

[42] Cerf A, Cau JC, Vieu C, Dague E. Nanomechanical Properties of Dead or Alive Single-Patterned Bacteria. Langmuir. 2009;25(10):5731–6. 10.1021/La9004642. WOS:000266081000045. 19334742

[43] Kumar A, Mortensen NP, Mukherjee PP, Retterer ST, Doktycz MJ. Electric field induced bacterial flocculation of enteroaggregative Escherichia coli 042. Appl Phys Lett. 2011;98(25). Artn 253701 10.1063/1.3600648. WOS:000292039900071.

[44] Schaudinn C, Stoodley P, Hall-Stoodley L, Gorur A, Remis J, Wu S, et al Death and Transfiguration in Static Staphylococcus epidermidis Cultures. Plos One. 2014;9(6). ARTN e100002 10.1371/journal.pone.0100002. WOS:000338709500026. 24964210 PMC4070908

[45] Wingender J, Neu TR, Flemming H-C. What are Bacterial Extracellular Polymeric Substances? Microbial Extracellular Polymeric Substances: Springer; 1999.

[46] Hashin Z, Shtrikman S. A Variational Approach to the Theory of the Elastic Behaviour of Multiphase Materials. J Mech Phys Solids. 1963;11(2):127–40. 10.1016/0022-5096(63)90060-7. WOS:A1963WZ82300005.

[47] Li SF, Liu WK. Numerical simulations of strain localization in inelastic solids using mesh-free methods. Int J Numer Meth Eng. 2000;48(9):1285–309. 10.1002/1097-0207(20000730)48:9<1285::Aid-Nme825>3.3.Co;2-8. WOS:000088002400002.

[48] Flemming HC, Neu TR, Wozniak DJ. The EPS matrix: The "House of Biofilm cells". J Bacteriol. 2007;189(22):7945–7. 10.1128/Jb.00858-07. WOS:000250991300001. 17675377 PMC2168682

[49] Costerton JW, Lewandowski Z, Debeer D, Caldwell D, Korber D, James G. Biofilms, the Customized Microniche. J Bacteriol. 1994;176(8):2137–42. WOS:A1994NG71700001. 8157581 10.1128/jb.176.8.2137-2142.1994 PMC205331

[50] Houry A, Gohar M, Deschamps J, Tischenko E, Aymerich S, Gruss A, et al Bacterial swimmers that infiltrate and take over the biofilm matrix. P Natl Acad Sci USA. 2012;109(32):13088–93. 10.1073/pnas.1200791109. WOS:000307551700053. 22773813 PMC3420162

[51] Wilking JN, Zaburdaev V, De Volder M, Losick R, Brenner MP, Weitz DA. Liquid transport facilitated by channels in Bacillus subtilis biofilms. P Natl Acad Sci USA. 2013;110(3):848–52. 10.1073/pnas.1216376110. WOS:000313909100022. 23271809 PMC3549102

[52] Shaw T, Winston M, Rupp CJ, Klapper I, Stoodley P. Commonality of elastic relaxation times in biofilms. Phys Rev Lett. 2004;93(9). Artn 098102 10.1103/Physrevlett.93.098102. WOS:000223555600067. 15447143

[53] Hassanpourfard M, Nikakhtari Z, Ghosh R, Das S, Thundat T, Liu Y, et al. Bacterial floc mediated rapid streamer formation in creeping flows. arXiv: preprint. 2015:1504.00098. 10.1038/srep13070 PMC4538384 26278133

[54] Buxton GA, Care CM, Cleaver DJ. A lattice spring model of heterogeneous materials with plasticity. Model Simul Mater Sc. 2001;9(6):485–97. 10.1088/0965-0393/9/6/302. WOS:000172464400002.

[55] Qi HJ, Ortiz C, Boyce MC. Mechanics of biomacromolecular networks containing folded domains. J Eng Mater-T Asme. 2006;128(4):509–18. 10.1115/1.2345442. WOS:000241820400007.

[56] Arruda EM, Boyce MC. A three-dimensional constitutive model for the large stretch behavior of rubber elastic materials. J Mech Phys Solids. 1993;41(2):389–412. 10.1016/0022-5096(93)90013-6

[57] Brown HR. A model of the fracture of double network gels. Macromolecules. 2007;40(10):3815–8. 10.1021/Ma062642y. WOS:000246280600045.

[58] Tanaka Y, Kuwabara R, Na YH, Kurokawa T, Gong JP, Osada Y. Determination of fracture energy of high strength double network hydrogels. J Phys Chem B. 2005;109(23):11559–62. 10.1021/Jp0500790. WOS:000229751800025. 16852418

