# Supplementary material for: Modeling of Mesoscale Variability in Biofilm Shear Behavior
Source: PLoS One. 2016 Nov 2;11(11):e0165593. doi: 10.1371/journal.pone.0165593 (PMC5091762; doi:10.1371/journal.pone.0165593)
Supplement: S1 File — This file contains detailed derivation of the digital biofilm model (DBM) and its grid independence study. (DOCX) [file pone.0165593.s001.docx]

Modeling of Mesoscale Variability in Biofilm Shear Behavior

(Supplementary information)

Pallab Barai^1*^, Aloke Kumar^2^ and Partha P. Mukherjee^1^

^1^Department of Mechanical Engineering, Texas A&M University, College Station, Texas, USA

^2^Department of Mechanical Engineering, University of Alberta, Edmonton, AB, Canada

In this supplementary information, detailed derivation of the digital biofilm model (DBM) and its grid independence study is provided.

**Present address:* Energy Storage and Distributed Resources Division, Lawrence Berkeley National Laboratory, Berkeley, California, USA

**1. COMPUTATIONAL METHODOLOGY ADOPTED IN**

**THE DIGITAL BIOFILM MODEL (DBM)**

Detailed description of the computational methodology adopted to capture the unfolding and rupture of eps matrix will be reported in this section. The digital biofilm model (DBM) consists of a network of lattice springs. The microstructure of a biofilm is superimposed on top of it. The bacterium and the eps matrix are assumed to display different elastic properties. Because of the unfolding of protein chains, stiffness of the lattice springs located within the eps matrix are assumed to increase with increasing amount of load. The lattice springs within the DBM network show only axial stiffness. To model the deformation of the spring elements, the equilibrium equation is solved:

$\frac{\partial{}_{t}^{t+\Delta t}\sigma_{ij}}{\partial{}_{t}x_{j}}=0$ (S.1.1)

Here, $t$ signifies the previous equilibrium step, $t+\Delta t$ corresponds to the current step which is an unknown and being solved for, $x_{j}$ signifies the spatial coordinates and $\sigma_{ij}$ indicates the stress tensor. Multiplying the above mentioned Eq. (S.1.1) with a small magnitude of virtual displacement and integrating over the entire volume of the element:

$\int_{{}_{t}V} \left(\frac{\partial{}_{t}^{t+\Delta t}\sigma_{ij}}{\partial{}_{t}x_{j}}\cdot\delta u_{i}\right)d{}_{t}V=0$ (S.1.2)

Here, ${}_{t}V$ is the volume of the element after the previous equilibrium step and $\delta u_{i}$ indicates the virtual displacement. Integrating Eq. (S.1.2) by parts, we get,

$\int_{{}_{t}V} \left({}_{t}^{t+\Delta t}\sigma_{ij}\cdot\frac{\partial\delta u_{i}}{\partial{}_{t}x_{j}}\right)d{}_{t}V-\int_{{}_{t}S} \left(\left({}_{t}^{t+\Delta t}\sigma_{ij}\cdot{}_{t}\hat{n}_{j}\right)\cdot\delta u_{i}\right)d{}_{t}S=0$ (S.1.3)

Here, $\hat{n}_{j}$ indicates the unit outward normal and ${}_{t}S$ signifies the surface of the domain of the previous equilibrium configuration. From the constitutive relations, the stress can be written in terms of the strain $\left({}_{t}^{t+\Delta t}\varepsilon_{ij}\right)$ and elastic modulus $\left(C_{ijkl}\right)$ as,

${}_{t}^{t+\Delta t}\sigma_{ij}=C_{ijkl}\cdot{}_{t}^{t+\Delta t}\varepsilon_{kl}$ (S.1.4)

Also, the traction force $\left({}_{t}\hat{t}_{i}\right)$ on the outward surface is defined as,

${}_{t}\hat{t}_{i}={}_{t}^{t+\Delta t}\sigma_{ij}\cdot{}_{t}\hat{n}_{j}$ (S.1.5)

Substituting Eq. (S.1.4) and (S.1.5) into the integral equation denoted as Eq. (S.1.3),

$\int_{{}_{t}V} \left(\left(\frac{\partial\delta u_{i}}{\partial{}_{t}x_{j}}\right)^{T}\cdot C_{ijkl}\cdot{}_{t}^{t+\Delta t}\varepsilon_{kl}\right)d{}_{t}V-\int_{{}_{t}S} \left({}_{t}\hat{t}_{i}\cdot\delta u_{i}\right)d{}_{t}S=0$ (S.1.7)

For a spring type geometric entity that spans only along the axial direction with only one degree of freedom at each node, the integral equation shown in Eq. (S.1.7) can be simplified into the following form:

$\int_{{}_{t}V} \left(\left(\frac{\partial\delta u_{1}}{\partial{}_{t}x_{1}}\right)\cdot C_{1111}\cdot{}_{t}^{t+\Delta t}\varepsilon_{11}\right)d{}_{t}V=\int_{{}_{t}S} \left({}_{t}\hat{t}_{1}\cdot\delta u_{1}\right)d{}_{t}S$ (S.1.8)

Since, one dimensional element has only two nodes, the previous expression can be simplified,

$\int_{{}_{t}V} \left(\left(\frac{\delta u_{1}^{2}-\delta u_{1}^{1}}{L}\right)\cdot C_{1111}\cdot\frac{{}_{t}^{t+\Delta t}u_{1}^{2}-{}_{t}^{t+\Delta t}u_{1}^{1}}{L}\right)d{}_{t}V={}_{t}\hat{t}_{1}^{2}\cdot\delta u_{1}^{2}-{}_{t}\hat{t}_{1}^{1}\cdot\delta u_{1}^{1}$ (S.1.9)

Here, $L$ signifies the length of the spring element at the end of the previous equilibrium step, and the superscript in the right top corner of each variable indicates the node number. The right hand side term is not integrated because it is just a point load, which is applied at the two ends of the one dimensional element. Assuming that the cross sectional area of the spring element is $A$ and $C_{1111}$ is the Young’s modulus for one dimensional spring element along the axial direction $\left(C_{1111}=E\right)$, Eq. (S.1.9) can be simplified and written in the matrix form as,

$\left[\begin{matrix} \delta u_{1}^{1} & \delta u_{1}^{2} \end{matrix}\right]\left[\begin{matrix} -1 \\ 1 \end{matrix}\right]\cdot E\cdot\left[\begin{matrix} -1 & 1 \end{matrix}\right]\left[\begin{matrix} {}_{t}^{t+\Delta t}u_{1}^{1} \\ {}_{t}^{t+\Delta t}u_{1}^{2} \end{matrix}\right]\cdot\frac{AL}{L^{2}}=\left[\begin{matrix} \delta u_{1}^{1} & \delta u_{1}^{2} \end{matrix}\right]\left[\begin{matrix} -{}_{t}\hat{t}_{1}^{1} \\ {}_{t}\hat{t}_{1}^{2} \end{matrix}\right]$ (S.1.10)

Cancelling the virtual displacement from both the left and right hand side, and realizing that the traction at both the nodes are equal in magnitude for a particular spring element,

$\frac{AE}{L}\left[\begin{matrix} 1 & -1 \\ -1 & 1 \end{matrix}\right]\left[\begin{matrix} {}_{t}^{t+\Delta t}u_{1}^{1} \\ {}_{t}^{t+\Delta t}u_{1}^{2} \end{matrix}\right]={}_{t}^{t+\Delta t}\hat{f}\cdot\left[\begin{matrix} -1 \\ 1 \end{matrix}\right]$ (S.1.11)

Here, the traction forces are assumed to be ${}_{t}\hat{t}_{1}^{2}={}_{t}\hat{t}_{1}^{1}={}_{t}^{t+\Delta t}\hat{f}$. For the one dimensional spring elements with two degrees of freedom at each node, the displacement and force vectors and the stiffness matrix can be written as,

${}_{t}^{t+\Delta t}u_{i}=\left[\begin{matrix} {}_{t}^{t+\Delta t}u_{1}^{1} \\ {}_{t}^{t+\Delta t}u_{2}^{1} \\ {}_{t}^{t+\Delta t}u_{1}^{2} \\ {}_{t}^{t+\Delta t}u_{2}^{2} \end{matrix}\right]$ and ${}_{t}^{t+\Delta t}f_{i}=\left[\begin{matrix} {}_{t}^{t+\Delta t}f_{1}^{1} \\ {}_{t}^{t+\Delta t}f_{2}^{1} \\ {}_{t}^{t+\Delta t}f_{1}^{2} \\ {}_{t}^{t+\Delta t}f_{2}^{2} \end{matrix}\right]$ and $k_{ij}=\left[\begin{matrix} E & 0 & -E & 0 \\ 0 & 0 & 0 & 0 \\ -E & 0 & E & 0 \\ 0 & 0 & 0 & 0 \end{matrix}\right]$

Here, ${}_{t}^{t+\Delta t}u_{i}$ is the local displacement vector, ${}_{t}^{t+\Delta t}f_{i}$ is the local force vector and $k_{ij}$ is the local stiffness matrix. Only the axial stiffness is taken into consideration here and the shear stiffness is neglected. To convert the force and displacement vectors from the local coordinate system to the global coordinate system, the vectors are multiplied with the transformation matrix $\left(T_{ij}\right)$:

$T_{ij}=\left[\begin{matrix} \cos\theta& \sin\theta& 0 & 0 \\ -\sin\theta& \cos\theta& 0 & 0 \\ 0 & 0 & \cos\theta& \sin\theta\\ 0 & 0 & -\sin\theta& \cos\theta\end{matrix}\right]$ (S.1.12)

Here, $\theta$ is the angle between the spring element and the global reference frame. Transformation of local displacement and force $\left({}_{t}^{t+\Delta t}f_{i}^{g}\right)$ vector and stiffness matrix into the global form $\left({}_{t}^{t+\Delta t}u_{i}^{g},{}_{t}^{t+\Delta t}f_{i}^{g},k_{ij}^{g}\right)$can be constructed as,

${}_{t}^{t+\Delta t}u_{i}^{g}=T_{ij}{}_{t}^{t+\Delta t}u_{j}$, ${}_{t}^{t+\Delta t}f_{i}^{g}=T_{ij}{}_{t}^{t+\Delta t}f_{j}$ and $k_{ij}^{g}=\left(T_{ij}\right)^{T}\cdot k_{ij}\cdot\left(T_{ij}\right)$ (S.1.13)

The increment in strain energy in each spring element is represented using the form:

$\Delta\psi=\frac{1}{2}{}_{t}^{t+\Delta t}u_{i}^{g}\cdot{}_{t}^{t+\Delta t}f_{i}^{g}=\frac{1}{2}{}_{t}^{t+\Delta t}u_{i}\cdot{}_{t}^{t+\Delta t}f_{i}$ (S.1.14)

Since the total strain energy is a scalar term, it does not matter whether the local or the global forms of the displacement and force vectors are used in the computation. The orthogonal transformation matrices generate an identity matrix when multiplied one with the transpose of the other. Updating the coordinate system happens after the system has reached equilibrium:

${}_{t+\Delta t}x_{i}={}_{t}x_{i}+{}_{t}^{t+\Delta t}u_{i}^{g}$ (S.1.15)

**2. STUDY OF MESH SIZE INDEPENDENCE FOR THE**

**DIGITAL BIOFILM MODEL (DBM)**

The theory described above have been applied to the DBM, which includes a network of lattice spring elements and a bacteria-substrate microstructure superimposed on top of it. The difference between extracellular-polymeric-substrate and the bacterium is taken into account by applying different magnitude of elastic modulus within the two regions. Stiffness of the lattice spring elements which lie at the interface is estimated based on weighted average scheme. Certain resolution of the lattice spring network is necessary to capture the biofilm microstructure.


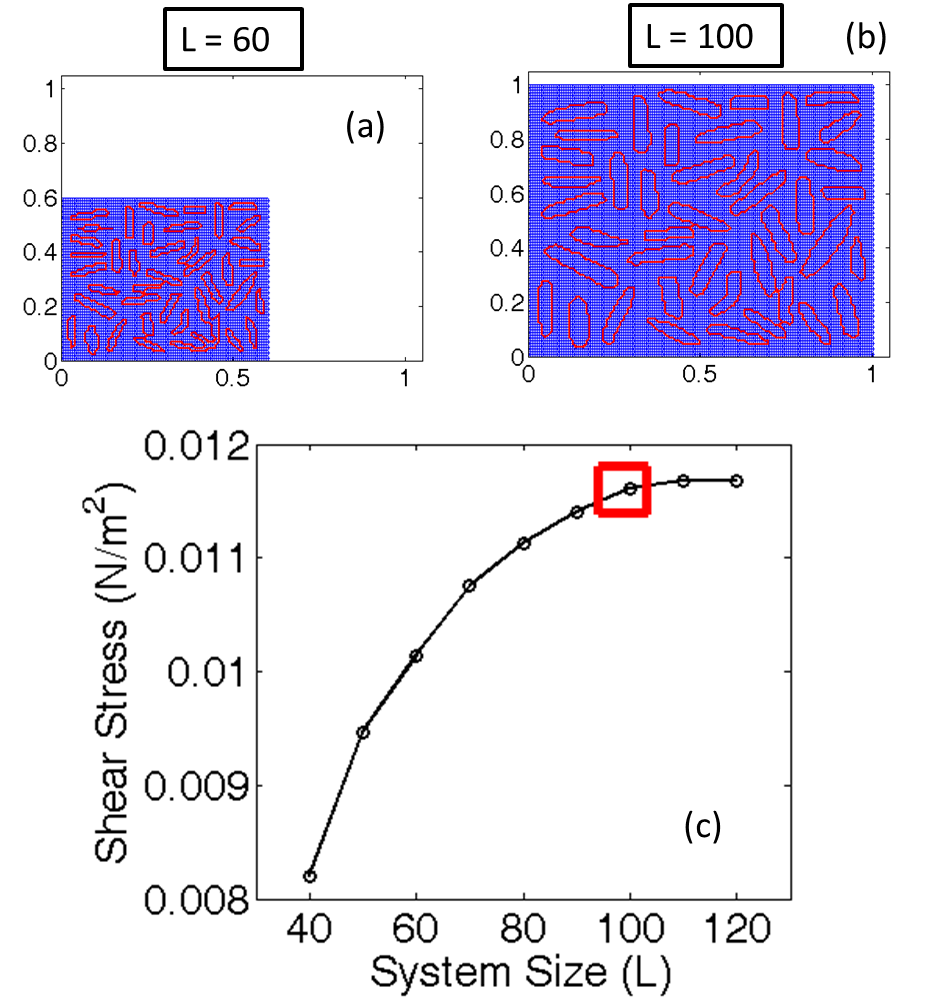


Fig S1. Demonstration of the lattice system size effect for the digital biofilm model (DBM). (a) Demonstration of the digitized biofilm domain with system size L = 60. (b) Digitized biofilm domain with system size L = 100. This system size has been adopted to run the final simulations. (c) Under the application of a fixed shear strain, the shear stress that developed within the lattice system of different size L = [40, 50, 60, 70, 80, 90, 100, 110, 120]. It is evident that with increasing system size, the shear stress converges to a fixed value. To keep the computational cost of running the simulations within limits, system size of L = 100 has been used to generate the final set of results.

Extremely small system size (L<60) may not be able to capture the microstructural details of the bacterium that is required to model the biofilm correctly. Sufficiently large systems (L≈80) capable of capturing microstructural details may also have numerical issues because a continuous media is being represented using a discrete system. To find the minimum system size that provides a consistent response against externally applied loads, a size dependence study is reported in Fig S1. Nine different system sizes have been considered to conduct the analysis (L = [40, 50, 60, 70, 80, 90, 100, 110, 120]). Shear strain of magnitude 0.01 is applied on top of the DBM. The bottom is kept fixed. The shear stress response from different sized systems has been reported. Once the internal shear stress becomes independent of the system size, that value of L can be taken as the optimum size. Fig S1(a) and S1(b) represents the DBM network on a normalized scale for L = 60 and L = 100, respectively. A biofilm microstructure with bacteria loading of 42% has been considered for this study. Fig S1(c) demonstrates the shear stress response obtained from all the nine different system sizes. It is evident that for system sizes L = 100 and beyond, the shear stress does not change with variation in system size. To keep the computational cost minimum without sacrificing accuracy, the system of size L = 100 has been considered for analysis purpose. All the simulation results reported in the main article used the DBM network of size L = 100.
